# Clinical utility of combined assessments of 4D volumetric perfusion CT, diffusion-weighted MRI and ^18^F-FDG PET-CT for the prediction of outcomes of head and neck squamous cell carcinoma treated with chemoradiotherapy

**DOI:** 10.1186/s13014-023-02202-x

**Published:** 2023-02-06

**Authors:** Hirokazu Tsuchiya, Munetaka Matoba, Yuka Nishino, Kiyotaka Ota, Mariko Doai, Hiroji Nagata, Hiroyuki Tuji

**Affiliations:** 1 Department of Radiology, Kanazawa Medical University, Daigaku 1-1, Uchinada, Kahoku, Ishikawa 920-0293 Japan; 2 Section of Radiological Technology, Department of Medical Technology, Kanazawa Medical University, Daigaku 1-1, Uchinada, Kahoku, Ishikawa 920-0293 Japan; 3 Department of Head and Neck Surgery, Kanazawa Medical University, Daigaku 1-1, Uchinada, Kahoku, Ishikawa 920-0293 Japan

**Keywords:** Head and neck cancer, Chemoradiotherapy, Prognostic prediction, Multiparametric imaging, Perfusion CT

## Abstract

**Background:**

Multiparametric imaging has been seen as a route to improved prediction of chemoradiotherapy treatment outcomes. Four-dimensional volumetric perfusion CT (4D PCT) is useful for whole-organ perfusion measurement, as it reflects the heterogeneity of the tumor and its perfusion parameters. However, there has been no study using multiparametric imaging including 4D PCT for the prognostic prediction of chemoradiotherapy. The purpose of this study was to determine whether combining assessments of 4D PCT with diffusion-weighted MRI (DWI) and ^18^F-fluorodeoxyglucose PET-CT could enhance prognostic accuracy in head and neck squamous cell carcinoma (HNSCC) patients treated with chemoradiotherapy.

**Methods:**

We examined 53 patients with HNSCC who underwent 4D PCT, DWI and PET-CT before chemoradiotherapy. The imaging and clinical parameters were assessed the relations to locoregional control (LRC) and progression-free survival (PFS) by logistic regression analyses. A receiver operating characteristic (ROC) analysis was performed to assess the accuracy of the significant parameters identified by the multivariate analysis for the prediction of LRC and PFS. We additionally assessed using the scoring system whether these independent parameters could have a complementary role for the prognostic prediction.

**Results:**

The median follow-up was 30 months. In multivariate analysis, blood flow (BF; *p* = 0.02) and blood volume (BV; *p* = 0.04) were significant prognostic factors for LRC, and BF (*p* = 0.03) and skewness of the ADC histogram (*p* = 0.02) were significant prognostic factors for PFS. A significant positive correlation was found between BF and BV (ρ = 0.6, *p* < 0.001) and between BF and skewness (ρ = 0.46, *p* < 0.01). The ROC analysis showed that prognostic accuracy for LRC of BF, BV, and combination of BF and BV were 77.8%, 70%, and 92.9%, and that for PFS of BF, skewness, and combination of BF and skewness were 55.6%, 63.2%, and 77.5%, respectively. The scoring system demonstrated that the combination of higher BF and higher BV was significantly associated with better LRC (*p* = 0.04), and the combination of lower BF and lower skewness was significantly associated with worse PFS (*p* = 0.004).

**Conclusion:**

A combination of parameters derived from 4DPCT and ADC histograms may enhance prognostic accuracy in HNSCC patients treated with chemoradiotherapy.

## Introduction

Head and neck squamous cell carcinoma (HNSCC) is the most common neoplasm of the upper aerodigestive tract in adults, and nearly two-thirds of patients with HNSCC present at a locally advanced stage. HNSCC has been managed primarily by surgery and/or radiotherapy with or without chemotherapy according to clinical staging, i.e., the Union for International Cancer Control TNM stage, but organ preservation strategies using concurrent chemoradiotherapy (CRT) have become an important treatment option for locally advanced HNSCC. However, the disease control achieved with CRT remains heterogeneous; treatment responses and clinical outcomes often differ among patients whose clinical staging and treatment method are the same. In addition, 15%–50% locoregional failure rates in the first 2 years after CRT have been reported in patients with advanced HNSCC [1]. The stratification of patients at risk of poor outcomes for CRT may therefore help avoid unnecessary treatment and facilitate the development of personalized management to improve clinical outcomes.

In the search for clinically convenient prognostic markers for the prediction of treatment outcomes of HNSCC patients treated with CRT, the utility of clinical variables (e.g., TNM stage or tumor volume) as well as functional imaging such as ^18^F-fluorodeoxyglucose (FDG) PET-CT, diffusion-weighted MRI (DWI), and dynamic contrast-enhanced MRI (DCE-MRI) has been widely investigated [2–4]. However, reliable imaging biomarkers for the estimation of prognosis have not yet been established.

Tumor tissue is known to be heterogeneous in proliferation, cellularity, and vascularity. Because of this functional and morphological heterogeneity of tumor tissues, the use of single-modality imaging limits the development of clinically useful prognostic imaging biomarkers. In order to evaluate tumor heterogeneity and its microenvironment for estimation of prognosis, improvement of imaging analysis methods and/or combined assessments of different functional imaging modalities have been studied [5, 6].

Perfusion CT (PCT) is able to probe the tumor microvascular environment by revealing the tumor vascular physiology and hemodynamic parameters; some previous studies have reported the utility of PCT for the prediction of treatment response to CRT in HNSCC patients [7–11]. However, these early PCT studies limited perfusion imaging to only a few slices covering 2–4 cm. Limited coverage resulted in evaluations of the perfusion parameters only at the level of the largest tumor diameter rather than the whole tumor volume. On the other hand, Rana et al. [12] investigated the prediction of treatment response to CRT in HNSCC patients using PCT with Z-axis coverage of 14 cm using the volume helical shuttle that enabled evaluation of entire tumor. However, there have been no PCT study evaluating perfusion of not only primary tumor but also cervical lymph nodes by the coverage of the whole neck (from the skull base to the thoracic inlet) for the prognostic prediction of CRT in HNSCC. The recent development of faster CT scanners has allowed wide z-axis coverage for PCT. PCT in adaptive 4D spiral mode entails state-of- the –art technology that enables whole-organ coverage in perfusion measurement [13]. The usefulness of adaptive 4D volume PCT (4D PCT) has been reported in the diagnosis of lung cancer [14], liver tumor [15], and cervical lymph node [16]. However, to our knowledge, there has been no study evaluating whether the combination of 4D PCT with other functional imaging modalities would improve estimations of prognosis in HNSCC patients treated with CRT.

The purpose of this study was to investigate the correlation of pretreatment 4D PCT, DWI, and PET-CT derived parameters with treatment outcome in HNSCC patients treated with CRT, and to determine whether the combined assessments of 4D PCT, DWI, and PET-CT for the primary tumor and metastatic lymph nodes improved the accuracy of long-term prognostic estimations in HNSCC patients treated with CRT.

## Materials and methods

### Patient population

This prospective study was performed with the approval of the local Medical Ethics Committee, and written informed consent for participation was obtained from all patients. Patients with histologically confirmed primary HNSCC who were planning to undergo definitive concurrent CRT at our institution were eligible. The inclusion criteria were as follows: newly diagnosed squamous cell carcinoma of the oropharynx, hypopharynx, or larynx; no positive HPV infection; no history of radiotherapy in the head and neck region; no history of anaphylactic reactions for iodinated contrast medium; performance status of 0–1 (Eastern Cooperative Oncology Group scale); age ≤ 80 years. The exclusion criteria were active invasive malignancy in the 3 years leading up to protocol entry, distant metastases, and serious medical complications, including active infectious disease, interstitial pneumonia, and cognitive dysfunction.

The study participants underwent pretreatment imaging examinations including 4D PCT, DWI, and PET-CT within the 5 weeks prior to the start of treatment.

### 4D PCT

All patients were scanned by a 64-MDCT dual-source scanner (SOMATOM® Definition Flash; Siemens, Erlangen, Germany); Volumetric PCT was used as the CT protocol. The scan range of the PCT was planned for coverage of the whole neck (from the skull base to the thoracic inlet). A total of 80 mL of nonionic iodinated contrast medium (iopromide 370; Bayer, Leverkusen, Germany) was injected via the antecubital vein using a fractioned injection protocol: 50 mL at 5 mL/s and 30 mL at 1 mL/s, followed by a 20-mL saline flush at 1 mL/sec. After a delay of 4 s, multiple scans within the defined scan range were obtained in 4D spiral mode. The PCT data acquisition was conducted as follows: 100 kV; 60–120 eff. mAs; detector configuration, 128 × 0.6 mm; rotation time, 0.28 s; slice thickness, 2 mm; four-dimensional range, 150–175 mm/1.5 s; first pass, 1.5 s × 30 cycles; delayed phase, 3.0 s × 10 cycles; total scanning time, 75 s.

All data sets were transferred to a workstation with commercially available PCT software. A regions of interest (ROI) was placed in the common carotid artery to generate the time-enhancement curve, and the perfusion data were post-processed by a deconvolution-based method. Two image sets were then reconstructed: [1] color-coded functional maps that represented the following perfusion parameters: blood flow (BF), blood volume (BV), permeability surface product (PS), and mean transit time (MTT), and [2] maximum intensity projections (MIPs) containing all arterial time points of the time-resolved raw data set, reconstructed using time-enhancement curves.

The ROIs were independently placed manually on the primary tumor and the largest metastatic lymph node on every slice of the MIPs by a radiologist with experience in PCT. For the ROI placements on the lesions, care was taken to include the solid portions and at the same time to exclude intra-tumor necrosis, surrounding blood vessels, and soft tissue. The ROIs placed on the MIPs were automatically copied to the color-coded functional maps, allowing for the assessment of corresponding quantitative perfusion values.

### DWI

MR imaging was performed using a 1.5-T system (Avant; Siemens) with a neck coil. The conventional MR imaging protocol consisted of axial T2-weighted images (turbo spin-echo sequence: TR/TE = 4000/90 ms, 512 × 256 matrix) and axial T1-weighted images (gradient recalled-echo sequence: TR/TE = 630/12 ms, 512 × 256 matrix). DWI was obtained with a single-shot spin-echo planar imaging sequence using a short inversion recovery time for fat suppression (TR/TE/TI = 4000/68/180 ms, 512 × 256 matrix). The sequence was repeated for three values of motion-probing gradients (b = 0, 90, and 800 s/mm^2^). Motion-probing gradients were placed on the three directions with the same strength. The field of view was 25 cm, and the slice thickness was 6 mm with an intersection gap of 3 mm. The apparent diffusion coefficient (ADC) was reconstructed for each pixel by the b-values of 90 and 800 s/mm^2^ using the standard software on the console.

The ROIs were manually drawn on the ADC_map_ by a radiologist with substantial experience of head and neck MR imaging. The ROIs were drawn on all imaging sections encompassing the solid portions of the primary tumors and the largest metastatic lymph node, and care was taken to exclude any obviously cystic or necrotic areas as well as surrounding blood vessels by referring to the T2- and T1-weighted images. The mean ADC values were then computed from the primary tumor and the metastatic lymph node, respectively. We performed a histogram analysis of ADC values to derive the parameters of kurtosis and skewness using offline software (Ziostation; Ziosoft, Tokyo).

### PET-CT

All patients fasted ≥ 6 h before the PET-CT examination. All patients’ plasma glucose levels were confirmed to be < 180 mg/dL. PET-CT (Biograph Sensation 16; Siemens) imaging was initiated 60 min after the intravenous injection of FDG (250–300 MBq). A whole-body PET emission scan was performed over the same area as that covered by CT with six bed positions. The protocol comprised an emission scan with 3 min/bed position. PET-CT images were analyzed on a dedicated workstation.

For the primary tumor and the largest metastatic node, the maximum standardized uptake value (SUV_max_), the peak standardized uptake value (SUV_peak_), the mean standardized uptake value (SUV_mean_), the metabolic tumor volume (MTV), and the total lesion glycolysis (TLG) were determined on PET images by a radiologist with substantial PET-CT experience. The tumor margins were identified on fused PET-CT images; a polygonal volume of interest, which included the entire lesion in the axial, sagittal, and coronal planes, was placed in the PET dataset (SUV_max_ threshold 40%). The SUV_peak_ was calculated as the average standardized uptake value within a 1 cm^3^ spherical volumetric ROI that included the maximum number of pixels. The MTV was defined as the total tumor volume with an SUV ≥ 2.5 and was calculated automatically. TLG was also automatically calculated by multiplying the MTV of the primary tumor by its SUV_mean_.

### Therapeutic regimen and follow-up

All patients were treated by intensity-modulated radiotherapy with 4-MV X-rays generated by a linear accelerator (Clinac iX; Varian Medical Systems, Palo Alto, CA). Radiotherapy was delivered 5 days/week using a 1-day fractionation of 2.0 Gy. The radiation doses administered to the primary tumor and the suspected metastatic lymph nodes were 70 Gy, while the dose of whole neck irradiation as prophylactic neck irradiation was 46 Gy. The concurrent chemotherapy consisted of weekly cisplatin (30 mg/m^2^) or cetuximab (initial loading dose of 400 mg/m^2^ followed by seven weekly infusions of 250 mg/m^2^).

The patients were followed up by clinical examinations every 1–3 months during the first year after the treatment, every 4 months for 2 years, and tapering to every 6–12 months thereafter. The post-treatment PET-CT to assess the treatment response was conducted at 8–12 weeks after the completion of CRT in each patient. In addition, contrast-enhanced CT or MRI was performed every 6 months as a routine follow-up examination or in the presence of clinical deterioration. Disease progression was defined as a persistent or recurrent primary lesion and/or metastatic lymph node and/or distant metastasis during the follow-up period consisting of either histopathological proof or clinically suspected recurrence resulting in a clinical assessment and an increase in the lesion size on serial CT or MRI examinations.

### Statistical analysis

The imaging parameters including BF, BV, MTT, and PS for PCT; ADC, kurtosis, and skewness for DWI; and SUV_max_, SUV_peak_, SUV_mean_, MTV, and TLG for PET-CT as well as other clinical parameters including patient age and sex, location of the primary tumor, TNM stage, chemotherapy regimen, and the tumor volume were evaluated as baseline variables. We performed logistic regression analyses to assess the relations of baseline variables to locoregional control (LRC) and progression-free survival (PFS). The optimal threshold of each imaging parameter, which showed the best trade-off between sensitivity and specificity for the survival rate observed in the entire study cohort, was determined by receiver operating characteristic (ROC) analysis [17]. All of the prognostic variables identified by univariate analysis were into the multivariate model. Multivariate analysis was performed using the Cox proportional hazards model with a forward selection procedure. Spearman rank correlation analysis was used to investigate the correlations among the variables. Additionally, ROC analysis was performed to assess the accuracy of the significant parameters for the prediction of LRC and PFS.

Regarding the parameters revealed to be significantly associated with LRC and PFS, we performed further assessments by the log-rank test among groups divided by the scoring system to determine whether these independent parameters could have a complementary role for the prognostic estimation of HNSCC patients.

Differences with a p-value < 0.05 were considered significant. All statistical analyses were carried out with JMP software package ver. 14.2 (SAS Institute, Cary, NC, USA).

## Results

Between September 2015 and March 2021, a total of 58 patients who met the inclusion criteria were enrolled in the study. Five of these patients were excluded from the data analysis: three for whom the image quality of PCT or DWI was poor due to artifacts of dental treatment, and two who discontinued treatment due to adverse events of CRT. A final total of 53 patients with squamous cell carcinoma of the oropharynx (n = 14), hypopharynx (n = 26), and larynx (n = 13) were eligible for the present analysis, including 11 females and 42 males; median age, 68.0 years. The patients' characteristics are summarized in Table 1.

**Table 1:** The patient’s characteristics

Characteristics	No. of patients (n = 53)
Age	
Median (range)	68.0 (47–79)
Female/Male	11/42
Tumor location	
Oropharynx	14
Hypopharynx	26
Larynx	13
T stage	
T1/T2/T3/T4	7/18/17/11
N stage	
N0/N1/N2/N3	26/8/14/5
Stage	
II/III/IVA/IVB	10/20/17/6

^*^All tumors were staged according to the 2017 UICC staging system

**Fig. 1:**
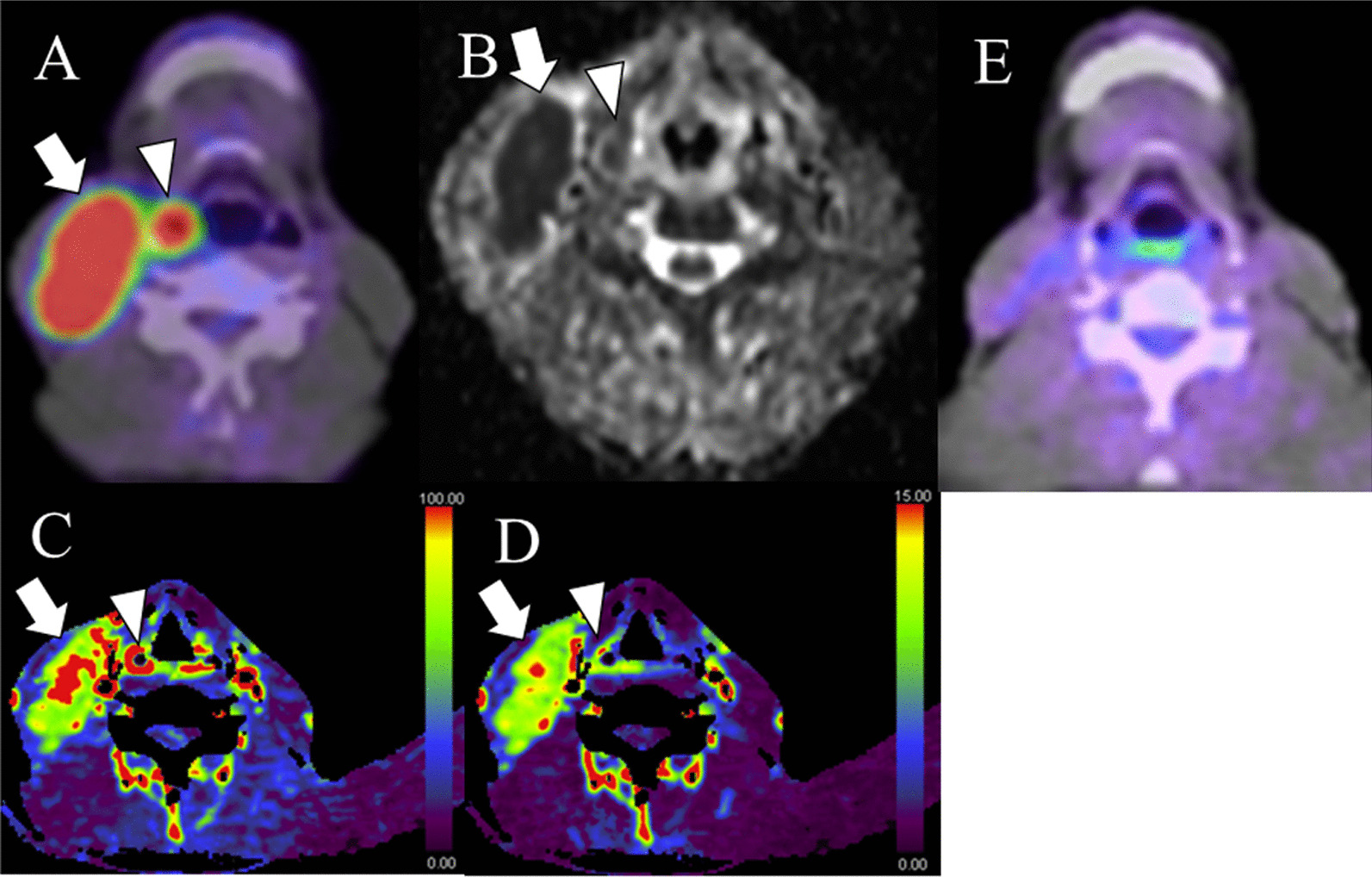
A 63-year-old man with right oropharyngeal squamous cell carcinoma (arrowhead) as well as enlarged lymph node (arrow) in level II on right side. **a** Pretreatment PET-CT. **b** The corresponding ADC map. **c** The corresponding BF map. **d** The corresponding BV map. The primary tumor showed BF value of 126.7 mL/min/100 mL, BV value of 12.3 mL/100 mL, and skewness of ADC of 0.71. The metastatic node showed BF value of 119.4 mL/min/100 mL, BV value of 11.1 mL/100 mL, and skewness of ADC of 0.69. **e** PET-CT at 12 weeks post-treatment demonstrates the persistence of mild ^18^F-FDG uptake in the metastatic lymph node site. However, clinical and imaging follow up did not disclose any recurrence of primary tumor and lymph node

The median follow-up time was 30 months (range 5–65 months). Twenty-nine of the 53 patients (54.7%) achieved disease control (Fig. 1), and the remaining 24 patients (45.3%) had disease progression; five patients had only local recurrence, eight patients had only regional lymph node recurrence, four patients had only distant metastasis, three patients had local recurrence and distant metastasis, and four patients had regional lymph node recurrence and distant metastasis. Forty (75.5%) patients were alive and thirteen (24.5%) had died during follow-up, all death being related to HNSCC. In the Kaplan–Meier analysis, the 1-, 2-, and 3-year rates for LRC were 72.3 ± 7.9%, 64.0 ± 8.9%, and 59.1 ± 9.5% while those for PFS were 69.3 ± 8.1%, 51.8 ± 9.1%, and 38.3 ± 9.6%, respectively.

**Fig. 2:**
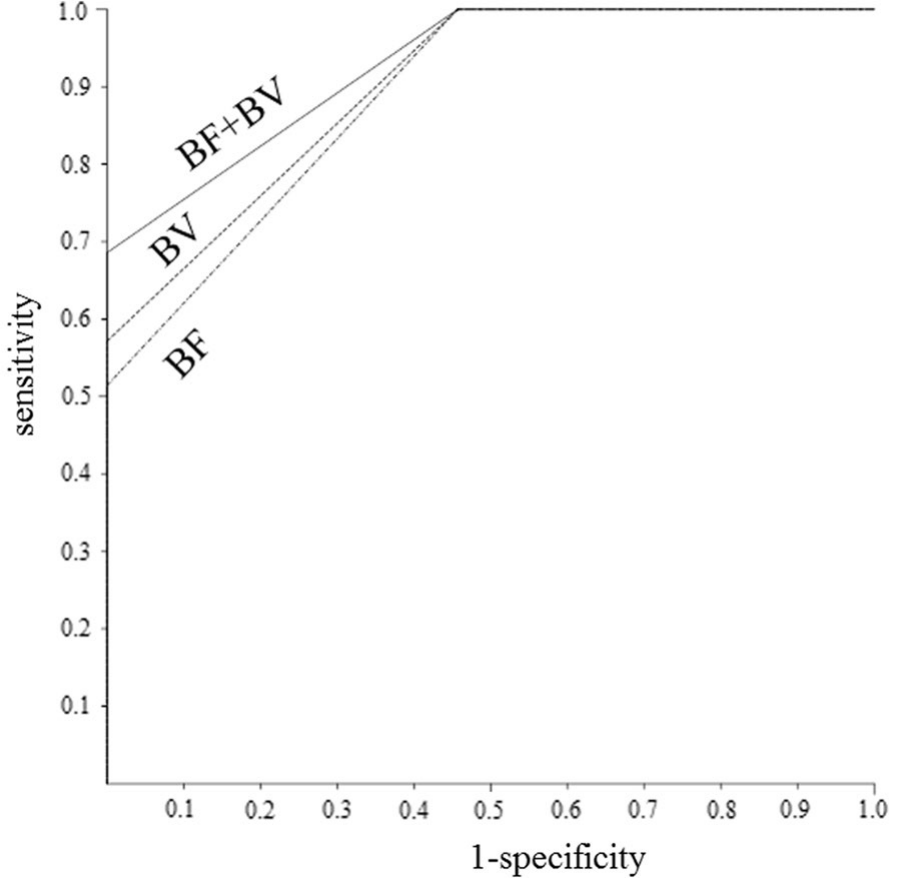
ROC curves demonstrating the differentiation of the predicted probabilities of locoregional control for the significant predictors obtained from imaging parameters

**Fig. 3:**
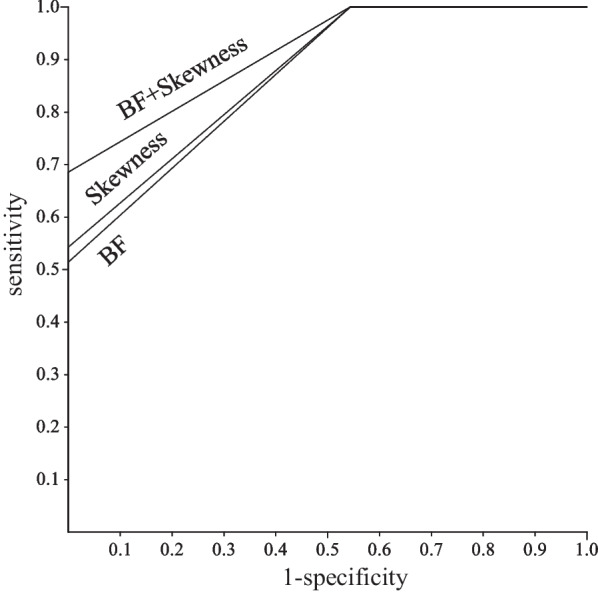
ROC curves demonstrating the differentiation of the predicted probabilities of progression-free survival for the significant predictors obtained from imaging parameters

**Fig. 4:**
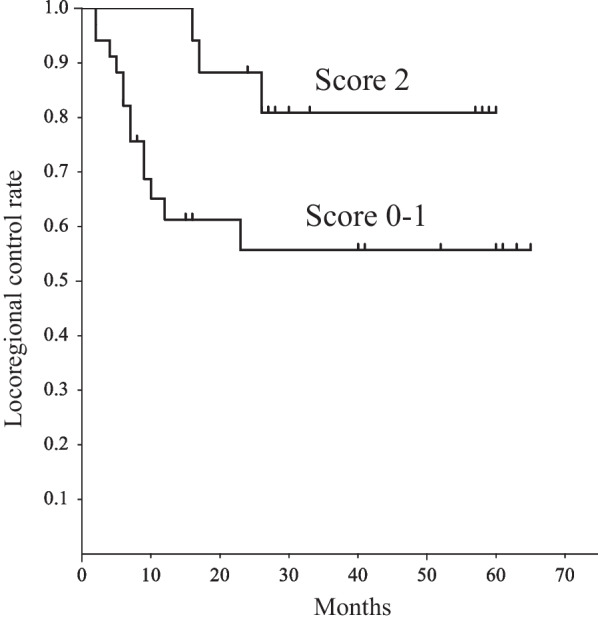
The comparison of Kaplan–Meier curves of locoregional control rate according to the scoring system

**Fig. 5:**
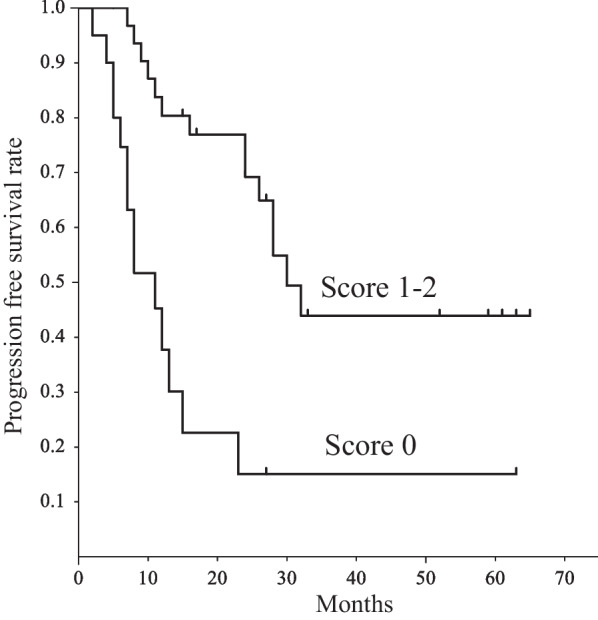
The comparison of Kaplan–Meier curves of progression-free survival rate according to the scoring system

Univariate and multivariate analyses were carried out to identify significant prognostic factors for LRC and PFS (Table 2). The univariate analysis showed that BF (*p* = 0.002), BV (*p* = 0.004), ADC (*p* = 0.008), kurtosis (*p* = 0.001), and skewness (*p* = 0.002) of primary tumor, and BF (*p* = 0.008), ADC (*p* = 0.01), and MTV (*p* = 0.04) of metastatic node were significant predictors of LRC. BF (*p* = 0.002), ADC (*p* = 0.01), kurtosis (*p* = 0.03), and skewness (*p* = 0.009) of primary tumor, and BF (*p* = 0.02), and ADC (*p* = 0.03) of metastatic node were significant predictors of PFS. After allowance for potential confounders, the multivariate analysis showed that BF (*p* = 0.02) and BV (*p* = 0.04) of primary tumor were independent predictors of LRC. BF (*p* = 0.03) and skewness (*p* = 0.02) of primary tumor were independent predictors of PFS. Spearman rank correlation analysis showed significant positive correlation between BF and BV (ρ = 0.6, *p* < 0.001) and between BF and skewness (ρ = 0.46, *p* < 0.01). The regression analysis for BF yielded a correct predicting of LRC of 77.8% and an ROC area under curve (AUC) of 0.89; BV yielded a correct prediction of 70% and an AUC of 0.9; and using both BF and BV yielded a correct prediction of 92.9% and an AUC of 0.93 (Fig. 2). Therefore, the combination of BF and BV tended to be superior to each of them for the prediction of LRC. The regression analysis for BF yielded a correct predicting of PFS of 55.6% and an AUC of 0.87; skewnwss yielded a correct prediction of 63.2% and an AUC of 0.88; and using both BF and skewness yielded a correct prediction of 77.5% and an AUC of 0.91 (Fig. 3). Therefore, the combination of BF and skewness tended to be superior to each of them for the prediction of PFS. Then, we further assessed a scoring system that summed up scores of 0 or 1 assigned to values below or above the thresholds of parameters using the Kaplan–Meier method and log-rank tests. The scores for LRC were as follows: 0 for BF ≤ 111.91 mL/min/100 mL and 1 for BF > 111.91 mL/min/100 mL; 0 for BV ≤ 9.42 mL/100 mL and 1 for BV > 9.42 mL/100 mL. The LRC rate of the patients with a score of 2 was significantly better than that of the patients with a score of 1 or 0 (*p* = 0.04) (Fig. 4). The scores for PFS were as follows: 0 for BF ≤ 111.91 mL/min/100 mL and 1 for BF > 111.91 mL/min/100 mL; 0 for skewness ≤ 0.63 and 1 for skewness > 0.63. The PFS of patients with scores of 0 was significantly worse than that of patients with scores of 1 or 2 (*p* = 0.004) (Fig. 5).

**Table 2:** Univariate and multivariate analysis of baseline variables for LRC and PSF

Characteristics ( threshold value)	Univariate (* p* value)		Multivariate (* p* value: HR (95%CI))	
	LRC	PFS	LRC	PFS
Age (68)	NS	NS		
Hypopharynx/others	NS	NS		
Stage: II, III/IVA, IVB	NS	NS		
CDDP/cetuximub	NS	NS		
Primary tumor				
BF (111.91)	0.002	0.002	0.02: 1.86 (1.36, 2.56)	0.03: 2.05 (1.42, 3.05)
BV (9.42)	0.004	NS	0.04: 2.08 (1.35, 3.11)	
MTT (5.92)	NS	NS		
PS (4.15)	NS	NS		
ADC_mean_ (1.17)	0.008	0.01	NS	NS
Kurtosis (3.3)	0.001	0.03	NS	NS
Skewness (0.63)	0.002	0.009	NS	0.02: 1.99 (1.22, 3.11)
SUV_max_ (11.13)	NS	NS		
SUV_peak_ (8.63)	NS	NS		
SUV_mean_ (6.94)	NS	NS		
MTV (5.22)	NS	NS		
TLG (30.35)	NS	NS		
Volume (9.67) Metastatic node	NS	NS		
BF (93.78)	0.008	0.02	NS	NS
BV (9.66)	NS	NS		
MTT (6.76)	NS	NS		
PS (3.79)	NS	NS		
ADC_mean_ (1.92)	0.01	0.03	NS	NS
Kurtosis (3.45)	NS	NS		
Skewness (0.87)	NS	NS		
SUV_max_ (10.05)	NS	NS		
SUV_peak_ (6.21)	NS	NS		
SUV_mean_ (5.12)	NS	NS		
MTV (5.23)	0.04	NS	NS	
TLG (28.34)	NS	NS		
Volume (4.78)	NS	NS		

*NS* no significance

## Discussion

Tumors easily become hypoxic and necrotic because of altered vascular architecture, rapid proliferation, and insufficient blood supply as the tumor rapidly grows. Tumors adapt to hypoxia by promoting angiogenesis, leading to higher perfusion than in other tissue [18]. Several studies have shown that tumor angiogenesis as measured by intratumor microvessel density has associations with an increased risk of locoregional recurrence, distant metastasis, and decreased patient survival [19]. Furthermore, Ach et al. reported that BF and BV derived from PCT in HNSCC patients correlated positively with microvessel density, indicating the potential of PCT for assessing angiogenesis [20]. On the other hand, regarding the relationship between PCT and treatment outcomes after CRT in HNSCC patients, Bisdas et al. [8] reported that BF and PS have a significant predictive value for local tumor control after CRT. Zima et al. [9] reported a linear relationship between high baseline BV values and tumor response after neoadjuvant chemotherapy. In addition, Truong et al. [10] ported a positive correlation between high baseline BF values and long-term LRC, consistent with the findings reported by Hermans et al. [11]. Therefore, CTP is thought to be a useful functional imaging biomarker enabling prediction of treatment response and LRC after CRT by monitoring tumor angiogenesis. However, early PCT studies were limited in terms of their z-axis coverage and resulted in evaluations of the perfusion parameters only at the level of the largest tumor diameter. Therefore, tumor heterogeneity and its perfusion parameters might not have been adequately taken into account. However, in this study, we could evaluate perfusion parameters throughout the whole tumor region and affected neck lymph nodes using volumetric PCT. As our results, BF and BV in primary tumor were found to be significant predictors of LRC, and the combined assessment of BF and BV was demonstrated to provide more accurate information for predicting LRC; for instance, the LRC rate of patients with both higher BF and higher BV was significantly better than that of the patients with only higher BF or only higher BV, or both lower BF and BV. Concerning the usefulness of the combined assessment of BF and BV, Bisdas et al. [8] reported the feasibility of visual evaluation of BF-BV mismatch for the prediction of long-term local tumor control after CRT as a possible indicator of tumor heterogeneity and increased angiogenesis. Their result was comparable with our result that reported BF and BV had complementary roles for the prediction of LRC. Consequently, our results suggested that PCT needs to be assessed volumetrically in view of tumor heterogeneity, and the combined assessment of BF and BV in primary tumor derived from volumetric PCT, which reflects tumor angiogenesis, may be a powerful predictor of LRC.

Tumor hypoxia is a major metabolic biomarker associated with increased resistance to CRT and inferior outcomes [21]. In a recent study evaluating the correlation of multiparametric MRI with FMISO PET-CT based hypoxic tumor volume in HNSCC, hypoxic regions were characterized by lower ADC and reduced perfusion K^trans^ and interstitial space volume Ve compared to non-hypoxic regions [22]. Namely, it was suggested that high tumor cellular density and reduction of interstitial space volume cause impairment of microcirculation, resulting in tumor hypoxia. In addition, in the study of prognostic estimation using combinations of DWI and DCE-MRI parameters in HNSCC patients treated with CRT, the factors of ADC and perfusion K^trans^ were found to be synergistic for the prediction of treatment response and long-term survival [23]. In short, these previous studies indicated that hypoxic tumors can be regarded as either diffusion- or perfusion-limited hypoxia or both, and the combined assessment of these imaging parameters may be useful for the prediction of treatment response and survival in HNSCC patients treated with CRT. In this study, skewness of the ADC histogram and BF in primary tumor were significant predictors of PFS. Furthermore, the combined assessment of skewness and BF was demonstrated to provide more accurate information in terms of predicting PFS; namely, the PFS rate of patients with both lower skewness and lower BF was significantly worse than that of patients with either higher skewness or higher BF, or both higher skewness and higher BF. Therefore, our results seemed to be comparable with previous studies that revealed the utility of combined assessment of diffusion and perfusion parameters for the treatment response and survival after CRT. However, to our knowledge, there has been no report that skewness derived from ADC histogram analysis is a significant predictor of PFS and has a complementary role with BF derived from PCT for proving more accurate information in terms of predicting PFS.

It is well known that ADC is associated with cellularity and/or proliferation in tumors. Some previous studies have shown that ADC is a useful imaging biomarker for the prediction of the treatment response to CRT in HNSCC [4]. However, HNSCCs are very heterogeneous in nature, with marked variations in proliferation and cellular differentiation in different regions of the tumors. Thus, ADC is thought to be insufficient to represent the full spectrum of histology within a tumor. In this study, skewness of the ADC histogram was a significant predictor of PFS, but not ADC_._ An ADC histogram analysis that better reflects the variable components and heterogeneity within the tumor may provide more information than the ADC [24]_._ In addition, studies that included an ADC histogram analysis have reported that skewness and kurtosis were significant parameters associated with the prediction of treatment response to CRT in HNSCC patients [25]. Skewness reflects an asymmetric distribution of the histogram attributed to heterogeneous structural and functional characteristics, i.e., intratumor heterogeneous regions of necrosis and hypoxia [24]. Therefore, although DWI is thought to be a useful imaging biomarker for the prognostic estimation after CRT, ADC histogram analysis, which reflects intratumor heterogeneity, may be required to predict treatment outcomes in HNSCC patients treated with CRT.

There have been a few studies examined whether multimodality imaging data from metastatic node could be used to predict the treatment response to CRT in HNSCC.

Jansen et al. [26] reported that pretreatment K^trans^ of DCE-MRI and SUV_mean_ in metastatic nodes were useful parameters for the prediction of short-term response to CRT. Ng et al. [27] reported that V_e_ of DCE-MRI and ADC in metastatic nodes as well as hemoglobin level were independent pretreatment prognostic parameters for the neck control after CRT. In addition, Kim et al. [28] reported that pretreatment ADC and change in ADC within the first week of treatment in metastatic nodes were useful parameters for the prediction of response to CRT. However, in these previous studies, the estimation of primary tumor using multimodality imaging has not been performed. In the current study, BF and ADC in metastatic node as well as BF, ADC, kurtosis, and skewness in primary tumor were significantly associated with both LRC and PFS in univariate analysis, however, metastatic node parameters did not retain their independent prognostic significance in multivariate analysis. Therefore, the evaluation of primary tumor using multimodality imaging was thought to be important to predict the treatment outcome after CRT. However, the different tissue characteristics between primary tumor and affected nodes might require separate evaluations in the assessment of perfusion and microenvironment of the two entities.

In our present patient series, PET-CT did not demonstrate a significant association with treatment outcomes. However, many studies have reported the utility of PET-CT for the prediction of treatment outcomes of HNSCC patients treated with CRT [29]. High tumoral metabolism such as high SUV_max_, SUV_peak_, MTV, and TLG was found to be correlated with the overexpression of HIF-1a, which characterize cellular response to hypoxic stress and is reported to be a prognostic factor for locoregional failure and disease free survival [30, 31]. In addition, enhanced metabolic activity reflects a tumoral phenotype that is prone to metastasize, which is reported to be correlated with distant metastasis free survival [32]. On the contrary, several studies have shown that semiquantitative evaluation for ^18^F-FDG-uptake parameters assessed by the SUV was not a useful prognostic factor for treatment outcomes [2]. In addition, Koyasu et al. [33] reported that qualitative uptake patterns of ^18^F-FDG (such as a ring-shaped uptake pattern) can provide better prognostic information than quantitative parameters. On the other hand, there have been many studies pointed out that PET-CT can reflect the early tumor metabolic changes during treatment, and changes in the metabolic activity of lesions may have an impact on prognosis in HNSCC patients treated with CRT [34]. Therefore, the prognostic significance of PET-CT quantitative parameters may remain controversial, however, the evaluation of changes in the metabolic activity during treatment may be necessary to clarify the significance adding PET-CT to multimodality imaging for the prognostic prediction after CRT in future studies.

Our study has some limitations. The results are from a single-institution analysis with a small number of patients. Our cohort was not homogeneous with regard to tumor locations. This limited our ability to report and capture the results of patients who met our eligibility criteria. However, our findings demonstrated the viability of the combination of DWI and PCT for the prediction of treatment responses and outcomes. It is thus worthwhile to conduct further studies with larger cohorts without potential selection bias. Another potential study limitation is that PCT acquisition involves a substantial radiation burden to patients. Although most published PCT studies of HNSCC report the use of 120 kV with 60 mA, a lower kilovoltage setting of 80–100 kV has been used in recent PCT studies to minimize radiation [12, 16]. In the PCT applied herein, 100 kV with 60–120 eff. mA was used to reduce the radiation dose. Further reduction of the radiation dose for whole-neck PCT while maintaining image quality is an important goal.

## Conclusion

The pretreatment combined assessments of BF and BV in primary tumor derived from 4DPCT may be powerful predictors of LRC, and BF in combination with skewness of ADC histogram analysis in primary tumor may provide a more accurate method for predicting PFS in HNSCC patients treated with CRT. The parameters derived from 4DPCT, which reflect the heterogeneity of the tumor and its perfusion, may be important factors for the prognostic prediction. Our results may be of great help in the selection of suitable HNSCC patients for CRT.

## Data Availability

Research data in this study are available on request from the corresponding author.

## Abbreviations

HNSCC: Head and neck squamous cell carcinoma
CRT: Chemoradiotherapy
4D PCT: Four-dimensional volumetric perfusion CT
BF: Blood flow
BV: Blood volume
PS: Permeability surface product
MTT: Mean transit time
MIPs: Maximum intensity projections
ROI: Regions of interest
DWI: Diffusion-weighted MRI
ADC: Apparent diffusion coefficient
FDG: Fluorodeoxyglucose
PET-CT: Positron emission tomography-CT
SUV: Standardized uptake value
MTV: Metabolic tumor volume
TLG: Total lesion glycolysis
DCE-MRI: Dynamic contrast-enhanced MRI
LRC: Locoregional control
PFS: Progression-free survival
ROC: Receiver operating characteristic
